# Examining chronic kidney disease screening frequency among diabetics: a POMDP approach

**DOI:** 10.1007/s10729-024-09677-4

**Published:** 2024-06-05

**Authors:** Chou-Chun Wu, Yiwen Cao, Sze-chuan Suen, Eugene Lin

**Affiliations:** 1 Daniel J. Epstein Department of Industrial and Systems Engineering, University of Southern California, Los Angeles, CA USA; 2 Department of Medicine, Division of Nephrology, University of Southern California, Los Angeles, CA USA; 3 Leonard D. Schaeffer Center for Health Policy & Economics, University of Southern California, Los Angeles, CA USA

**Keywords:** POMDP, Disease screening, Chronic kidney disease, Diabetes, Proteinuria, Operations research, Simulation, Markov Decision Process

## Abstract

Forty percent of diabetics will develop chronic kidney disease (CKD) in their lifetimes. However, as many as 50% of these CKD cases may go undiagnosed. We developed screening recommendations stratified by age and previous test history for individuals with diagnosed diabetes and unknown proteinuria status by race and gender groups. To do this, we used a Partially Observed Markov Decision Process (POMDP) to identify whether a patient should be screened at every three-month interval from ages 30-85. Model inputs were drawn from nationally-representative datasets, the medical literature, and a microsimulation that integrates this information into group-specific disease progression rates. We implement the POMDP solution policy in the microsimulation to understand how this policy may impact health outcomes and generate an easily-implementable, non-belief-based approximate policy for easier clinical interpretability. We found that the status quo policy, which is to screen annually for all ages and races, is suboptimal for maximizing expected discounted future net monetary benefits (NMB). The POMDP policy suggests more frequent screening after age 40 in all race and gender groups, with screenings 2-4 times a year for ages 61-70. Black individuals are recommended for screening more frequently than their White counterparts. This policy would increase NMB from the status quo policy between $1,000 to  $8,000 per diabetic patient at a willingness-to-pay of $150,000 per quality-adjusted life year (QALY).

## Highlights

- To encourage timely diagnosis of chronic kidney disease (CKD) among diabetics, we developed screening guidelines stratified by age, race, gender, and prior testing results using a POMDP to identify the frequency at which a patient should be screened.
- We solved for the POMDP CKD screening policy using nationally representative empirical data to provide age-specific screening guidelines.
- Results suggest that diabetic individuals should be offered screening more often than recommended by the status quo.
- We identified a CKD screening policy that would generate a higher net monetary benefit (NMB) by roughly $1,000-$8,000 per individual with diabetes in the US above age 30 compared to the status quo.

## Introduction

### Background

Chronic kidney disease (CKD) describes the gradual loss of kidney function, defined as kidney damage that persists for at least three months. CKD can progress to advanced renal failure and end-stage renal disease (ESRD). ESRD is treated using dialysis, which requires a machine to replace native kidney function. According to the Centers for Disease Control and Prevention (CDC) and the National Institutes of Health (NIH), kidney disease was the ninth leading cause of death in the US in 2019 [1]. In 2018, approximately 40 million individuals, or 15% of adults in the U.S., suffered from CKD [51]. Of these individuals, 6% had CKD stage 3 or higher, and more than 785,000 of those lived with ESRD [51]. Early detection can help prevent the progression of kidney disease to kidney failure. However, 90% of adults with CKD are unaware of their health status [65].

CKD is not only a major cause of mortality but also a cause of excessive medical costs in the U.S., particularly for older adults. Costs of CKD rise rapidly with disease progression. Evidence from the medical literature suggests that the annual cost of CKD can range from $10,000 to $100,000 per patient, with the highest costs associated with ESRD [16, 29, 51]. Thus, early detection of CKD is essential to slow CKD progression and decrease potential medical spending.

The lifetime risk of developing CKD is high: previous studies estimate that 40% of US adults who do not yet have CKD at age 50 are likely to develop CKD within their lifetime [37]. Since clinical manifestations often occur in later CKD stages, nephrologists have come to rely heavily on biomarkers to diagnose CKD, mainly estimated glomerular filtration rate (eGFR) and proteinuria (protein in the urine) [14]. GFR is a key indicator of renal function. An estimated GFR (eGFR) is a mathematically derived value based on a patient’s GFR, serum creatinine level, age, race, and gender [40]. eGFR levels and proteinuria status can be used to determine the stage of a patient’s kidney disease.

There are documented risk factors for CKD. Persistent proteinuria is a key indicator of CKD and accelerates the speed of eGFR decline. Diabetic and elderly patients are at particularly high risk of CKD, and approximately 40% of adults with diabetes will develop kidney disease [24]. Furthermore, gender may play an important role. For example, men progress more quickly to ESRD than women [9]. Besides the above risk factors, there exist significant disparities in CKD rates and outcomes between Black and White Americans [11]. CKD incidence is higher among Black compared to White individuals, a difference that is explained in part by proteinuria risk [41]. Compared to White patients, Black patients are not only more likely to acquire CKD but also to die from CKD [41]. Efforts to identify and slow progression of CKD at earlier stages are needed to reduce racial disparities.

### Motivation

Screening and early treatment is a critical component of CKD care, as it has a long asymptomatic period, its health outcomes can be severe, and screening tests are available. The impact of CKD screening is potentially significant, as only 10% of individuals with CKD are aware of their true health state and there exist interventions proven to reduce the risk of CKD progression [15]. Improved screening strategies may therefore be useful for the effective prevention and management of CKD. However, prior cost-effectiveness work shows that only targeted screening is societally beneficial. There is insufficient evidence to recommend routine screening for the general population in the US [7], and in fact the American College of Physicians (ACP) recommended against screening for chronic kidney disease in asymptomatic adults without risk factors for chronic kidney disease [52]. On the other hand, screening for proteinuria in individuals with diabetes, hypertension, or older age (55 or above) has been determined to be efficient and cost-effective in identifying those with CKD [7].

Screening for a disease will generally be more successful and cost-effective if the disease is relatively common among those targeted. Previous literature shows that screening for CKD can be cost-effective if high-risk groups are targeted (see [18, 31]). The National Kidney Disease Education Program suggests people with diabetes should be checked for kidney function once per year, which is also recommended by the NIH and the American Diabetes Association (ADA). However, whether this annual screening is optimal is still debatable since physicians often suggest more frequent screening [21, 57].

Given this controversy, in this research we focus on CKD screening among people who have diagnosed diabetes. This is an important patient population for CKD control, as nearly half of type 2 diabetics with CKD are unaware of their CKD state [6]. In this work, we identify patient-specific guidelines that account for a patient’s age, gender, race and health information revealed over time using a POMDP framework. Our work provides the following contributions to the literature: (1) We carefully design and parameterize a POMDP model of CKD among diabetics in the US using the medical literature, a microsimulation model, and oversight from a medical expert; and (2) We use the microsimulation model of CKD to understand POMDP policy impacts on health outcomes in the diabetic population and provide more interpretable and implementable age- and race-specific screening guidelines. To our knowledge, there has been no prior attempt to use such a detailed microsimulation model for CKD to inform POMDP inputs for the purposes of creating demographically-specific screening guidelines for the vulnerable diabetic population. The microsimulation draws on nationally-representative data to generate POMDP inputs (rewards, transition probabilities, etc.). This is useful since we parameterize the POMDP by race, gender, age, proteinuria status, treatment status, as well as CKD states; a microsimulation can conveniently provide lifetime health trajectories for any of these population groups in a standardarized way. After solving the POMDP, we use the microsimulation to translate the belief-based POMDP policy into interpretable guidelines for clinical use.

## Literature review

### Prior use of POMDPs in health care applications

Many medical decisions hinge on estimates of disease progression over time with multiple risk factors. These decisions may be informed by POMDPs. POMDPs are a class of widely used dynamic stochastic models that allow for repeated decision making with uncertain sensing. This type of model can be used to capture the probabilistic progression of disease over time and allows modelers to identify the preferred action to take while considering all uncertainty realizations. POMDPs have shown great potential in personalized medical recommendations through considering the benefits and harms of decisions under different risk profiles and perspectives. Recently, POMDPs have played an important role in healthcare research where previous studies have applied POMDPs extensively in policy modeling [25, 55]. In particular, many screening and treatment decision problems have been formulated using a POMDP framework where authors have focused on a variety of topics, including repeated screening decisions, medical initiation, organ transplantation, and return to play after concussion [2, 5, 17, 23, 33, 35, 50, 56, 66].

In particular, some works focus on developing personalized diabetes screening plans using POMDPs. For instance, [30] and [63] use POMDP frameworks to designed personalized diabetes screening plans. Ali Hajjar and Alagoz [3] extends these works by considering screening for breast cancer among diabetics. Similarly, we consider CKD screening for patients already diagnosed with diabetes. Ali Hajjar and Alagoz [3] primarily examines interactions between diseases and compares screening frequency outcomes between non-diabetics and diabetics.

By constrast, in this work, we focus on identifying screening recommendation differences between demographic groups among diabetics. To our knowledge, no work has previously addressed the problem of designing screening frequencies for CKD among diabetics using a POMDP framework. This work considers differences in CKD progression and mortality associated with race (White vs. Black), biological sex (male vs. female), and age. We also provide national-level guidelines that may be easier for clinicians to understand and implement.

POMDP outcomes may be difficult to interpret, as they rely on beliefs on a probability simplex, which may not conform with how a clinician thinks of patient health in practice. We use a microsimulation to identify an approximation of the POMDP policy by age, race, and gender characteristics to facilitate practical implementation. Prior work has also examined ways to better formulate POMDPs for increased transparency. For instance, [4] use a decomposition of the core state space into a tuple consisting of clinically relevant measures for breast cancer screening. This can be a useful way to represent the core state space for clinical interpretability. Others have used POMDPs and simulations together, as we do (see [17, 49] as examples). However, to the best of our knowledge, we are the first to do so in the context of CKD screening for diabetics.

### Prior CKD screening literature

CKD screening is recommended by several professional agencies and appropriate in at-risk asymptomatic individuals. For instance, selective screening is supported by the National Kidney Foundation (NKF), the Renal Physicians Association (RPA), and the ADA [8]. These organizations all support screening for individuals with diabetes. The NKF and RPA further recommend screening in other risk groups including Black patients and those 60 years or older. Moreover, the U.S. Preventive Services Task Force (USPSTF) suggests monitoring proteinuria and eGFR values for diabetics [39]. We therefore use an annual screening frequency for diabetic patients as the status quo policy in our numerical comparisons.

Most screening studies are based on cost-effectiveness analyses. One study suggests that general screening for CKD detection can be justified as cost-effective in a population with high prevalence of the disease such as in Japan (20%) and other Asian countries [31]. A Canadian study shows that screening for CKD in Canadian indigenous peoples is cost-effective as Canadian indigenous individuals have rates of kidney failure that are 2- to 4-fold higher than non-indigenous people [18]. Previous studies also suggest that screening among Black patients should be more cost-effective than non-Black patients [27, 61]. A US study supports annual CKD screening for patients with diabetes starting at age 50 using a willingness-to-pay threshold of $50,000/QALY gained [46]. However, although annual screening can be cost-effective, it may not be optimal in terms of maximizing expected discounted future net monetary benefit (NMB) for patients at earlier ages. Moreover, many physicians may suggest more frequent screening (every six months) for at-risk groups [21, 57]. These results tell us that screening programs can be cost-effective if high-risk groups are targeted.

While previous studies agree that CKD risk factors include diabetes, age, and African ethnic background, there exists ambiguity in the age interval of screening and only consider wide age intervals (such as from 0 to 54, and age 55 and above) [45]. In addition, like most screening recommendations, no current suggestions are based on optimization analyses and no papers have examined optimal CKD screening times. Instead, results derive from cost-effective analyses and simulations. This means that the identified policies, while cost-effective among the compared interventions, are likely to be suboptimal.

To address these issues, we used a POMDP to create nuanced guidelines to better inform physicians and patients of the optimal frequency to screen within different age intervals for White and Black race. To our knowledge, there is no work in the existing literature that considers screening frequency for CKD using POMDP models, particularly within the context of age, proteinuria, diabetes, race and previous test results.

## Methods

### Overview of problem structure

We use a POMDP framework to identify a better CKD screening policy by age, race, and gender. We parameterize the POMDP transition probabilities and terminal rewards using a microsimulation (see Appendix B), which allows us to accurately estimate disease progression as well as health and cost outcomes associated with treatment (reduction of annual eGFR decrements).

Our POMDP reflects the CKD screening problem in real world settings. Every three months, a diabetic patient has a routine screening with their physician to monitor their health, and they may undergo tests to determine whether they have developed CKD (eGFR $\le 60$ and/or developed persistent proteinuria). In our model, individuals without proteinuria may stochastically acquire proteinuria each period, and those with proteinuria are assumed to have proteinuria for the remainder of their lives. During the screening, the physician may ask the patient to undergo testing for CKD. To decide whether testing was needed, we assume that the physician estimates a patient’s belief state using a probabilistic distribution, then decides whether to recommend screening to maximize the patient’s expected future net monetary benefit, or NMB, a monetary health measure is often used in health policy making. We follow the health economics and medical decision making literature, which commonly uses willingness-to-pay for quality-adjusted life years as a method to convert health outcomes into NMB (see [32, 36, 38, 58, 62]). Specifically, the NMB is a function that considers health benefits (as measured through quality adjusted life years, QALYs, and translated into a dollar value by multiplying by a willingness-to-pay value, *WTP*) from which it subtracts costs, e.g., NMB = (QALYs) $\times$ (WTP)- (costs).

If a screening is requested, the physician will receive a perfect laboratory test result (both eGFR level and proteinuria status) that shows the patient’s CKD state at the next patient visit (so the test result reflects possible disease progression that occurred in those three months). This implies that we only focus on persistent proteinuria and this diagnostic process is 100% sensitive and specific for all CKD stages. While a single screening test in reality may result in a false-positive or a false-negative, it is likely that a false-positive test result would be corrected quickly given that additional testing is often performed once a patient is referred to a nephrologst for CKD, and even if not, the regular testing associated with CKD treatment would quickly catch the error. Conversely, a patient with CKD stage 3 or above may show symptoms that prompt re-testing if the first test results in a false-negative. As these events would typically take place within a single epoch of time, we assume sensitivity and specificity is effectively perfect in the model (and we vary sensitivity and specificity in sensitivity analyses). The physician can then update his or her estimate of a patient’s health state using his knowledge of CKD progression rates and this test result. This decision process is repeated every three months. This process is consistent with conventional POMDP processes (see [35, 50, 56]).

To create a race- and gender-specific policy, we assumed that the the physician’s knowledge of disease progression varies by age, race, gender, and proteinuria status. This allows the physician’s estimate of the patient’s disease progression risk to vary by age, race, and CKD stages. This knowledge is reflected in the POMDP transition matrices, which also vary by age, race, and CKD stages.

### Notation and POMDP model formulation

We formulate this CKD screening problem as a finite horizon problem with 3-month decision epochs, ${t}= \{1, 2,..., T = 221\}$ where ${t}= 1$ denotes the end of the first 3-month starting at age 30 with a final decision at age 84.75 (at time $T-1$). While the age of screening termination should be a shared decision between the physician and the patient, we need a fixed age to stop screening for use in our model. We therefore use age 85 as the termination age after consulting with our clinical expert (a nephrologist who treats CKD patients). We formulate our POMDP as a tuple $(\mathcal {S}, \mathcal {A}, \mathcal {P}, \mathcal {O}, \mathcal {R})$, which is defined as follows.

The core state space denotes the set of possible health states of the patient. Following prior literature, our CKD health states correspond to CKD stages (defined by eGFR thresholds) in combination with the presence or absence of proteinuria (see Table 1). We therefore define states $\mathcal {S} = \{1,2,3,4,5,6,7,8,9,10\}$ where 1 represents a patient with no CKD, 2 represents CKD stage 1 (with proteinuria), 3 represents CKD stage 2 (with proteinuria), 4 and 5 represent CKD stage 3 without and with proteinuria respectively, 6 and 7 represent CKD stage 4 without and with proteinuria respectively, 8 and 9 represent CKD stage 5 without and with proteinuria respectively, and 10 represents death. We do not distinguish between disease induced death states and non-disease induced death states, as disease-specific death probabilities are captured in the transition matrices. The health state at decision epoch *t* can be represented using $s^t$.

**Table 1:** Definition of CKD states from prior literature [26]

State	Kidney damage (Proteinuria)	eGFR
Normal	No	60+
CKD 1	Yes	90+
CKD 2	Yes	$60-89$
CKD 3	Yes or No	$30-59$
CKD 4	Yes or No	$15-30$
CKD 5	Yes or No	<15

The belief space is defined on $\varPi (\mathcal {S}) = \{ \pi \in \mathbb {R}^m:\sum _{i=1}^{m}\pi (i)=1,\ \pi (i)\ge 0 \ \forall i\}$ where *m* represents total number of states in $\mathcal {S}$. In our case, $\varPi$ is a $\mathbb {R}^{10}$ probability simplex. Each belief $\pi$ is a $1\times 10$ vector that contains the probability of being in each state. Each element in $\pi$ can be represented using $\pi (i)$ for $i = 1,2,3,4,5,6,7,8,9,10$. Then the belief at decision epoch *t* can be represented using $\pi ^t$ where $\pi ^t(i)$ represents the *i*th element of this belief.

The action space, $\mathcal {A} = \{ \textit{Screen} , \textit{Wait}\}$, denotes the feasible set of actions at each time epoch provided a patient is alive and not yet on CKD treatment. Individuals in the death state cannot be screened, and no further actions can be performed once any CKD stage is observed (the decision process stops if screening reveals a positive CKD test result, as the patient begins CKD treatment). The action at decision epoch *t* can be represented using $a^t$. We follow the guidelines proposed by KDIGO (Kidney Disease: Improving Global Outcomes), a working group which published updated guidelines on the detection, evaluation, classification, and management of CKD, to define CKD screening as a process that measures serum creatinine, estimates of GFR using a serum creatinine-based equation, measures the urine albumin/creatinine ratio, and performs urinalysis (see [22]). KDIGO guidelines are widely considered by nephrologists as the definitive set of guidelines for kidney disease. We assume that proteinuria and eGFR tests are always done concurrently (not in isolation), as this is typical clinical practice. In our model, we consider these two measures as a single screening procedure which results in an observation of CKD stage.

The set of core state transition probability matrices is represented using $\mathcal {P}$. Each element of the matrix $P^{t}$ is represented by $p_{ij}^{t}(Age, gender, Race, Proteinuria,$ *Treatment*), the probability of transitioning from health state *i* to *j* given risk factors age, gender, race, proteinuria status, and treatment status at decision epoch *t*. We refer to these demographic-specific values as $p_{ij}^{t}$ for simplicity hereafter. We assume proteinuria is persistent (once acquired, a patient has proteinuria until death) and receiving treatment will only slow down the eGFR progression rate. To model this, we apply a reduction factor to the annual eGFR decrement equation to reflect treatment effectiveness, which we then incorporate into our transition matrices. Treatment effectiveness ranges from 20% to 40%, and we use 30% as our base case value [26]. These matrices and their calculation are discussed further in Appendix Section D.

**Fig. 1:**
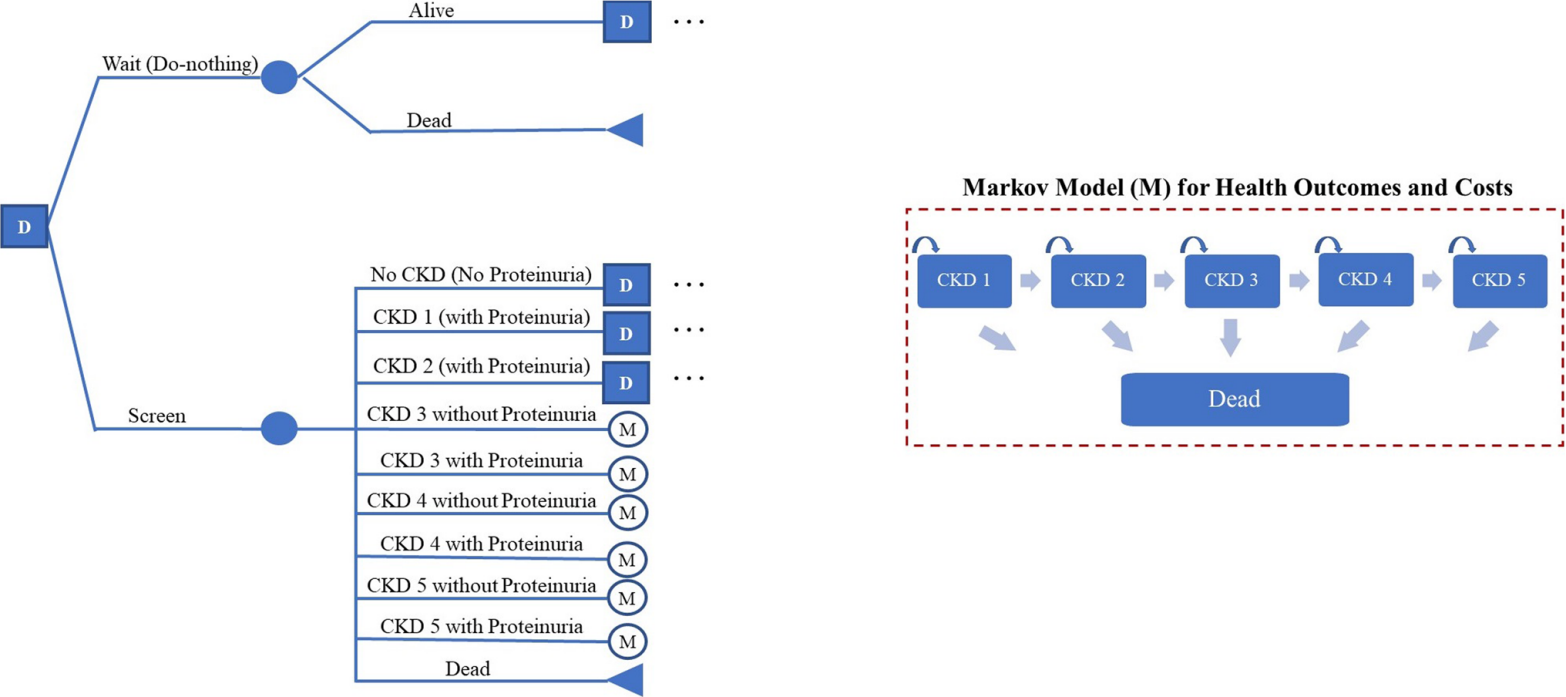
This decision process is repeated each three-month period from ages 30 to 85. The decision tree stops if a patient’s test result shows CKD 3 or above; we assume the patient is then placed on treatment. We estimate the remaining lifetime rewards associated with treatment using a Markov Model that estimates the post-treatment health outcomes and costs starting at that patient’s age. The decision tree terminates at age 85

The set of observation matrices is represented using $\mathcal {O}$. ${\textbf{O}}^{Screen}=\{1, 2, 3, 4, 5, 6, 7, 8, 9, 10\}$ denotes the set of possible observations after conducting the action (administering the CKD test) and is consistent with the core health states.

If the patient does not undergo screening, the physician can only observe whether the patient is still alive or has died before their next appointment. This decision process (see Fig. 1) repeats until the patient dies or tests positive for CKD stage 3 or above, upon which we assume the patient receives treatment and the decision process ends as no further screening is needed (in accordance with current drug therapy guidelines [19]). The POMDP policy is then a series of *screen* or *wait* actions over the time horizon that maximize the patient’s expected future NMB. The observation space for waiting is represented by ${\textbf{O}}^{Wait}=\{\textit{Alive}, \textit{Dead}\}$.

Observation probabilities are captured inside matrix ${O}^a$. Each element of the matrix is represented by $q_{jk}^{a}$, the probability of observing outcome *k* given state *j* and action ${a}$. We assume both sensitivity and specificity are 100% for all states. For action = *Screen*, we therefore have $q_{jk}^{Screen} = 1$ if $j = k$ and $q_{jk}^{Screen} = 0$ otherwise. The observation matrix for screening is therefore a ten by ten identity matrix.

In the case where no screening is performed, ${O}^{Wait}$ has the following observation probabilities. For $k = \textit{Alive}$, $q_{jk}^{Wait} = 1$ if $j = 1,2,...,9$ and $q_{jk}^{Wait} = 0$ otherwise. For $k = \textit{Dead}$, $q_{jk}^{Wait} = 1$ if $j = 10$ and $q_{jk}^{Wait} = 0$ otherwise.

After receiving observations (test results if the patient underwent screening, death status if not), the physician updates his or her belief of the patient’s health state. We capture this updated belief using Bayes’ rule:

1 $$\begin{aligned} \pi ^{t+1}(j)=\frac{\sum _{i \in \mathcal {S}} \pi ^t(i) p^{t}_{ij} q^{{a}}_{jk}}{\sum _{i \in \mathcal {S}} \sum _{l \in \mathcal {S}}\pi ^t(i) p^{t}_{il} q^{{a}}_{lk}}\ \end{aligned}$$

This equation is a function of current belief at time *t* ($\pi ^t$), transition probabilities $p^{t}_{ij}$, and observation probabilities $q^{a}_{jk}$, and provides the physician’s belief that the patient’s updated health is in state *j* given observation *k* and action *a*.

The set of stage rewards and terminal rewards is represented using $$\mathcal {R}$$. The stage reward at decision epoch *t* given belief $$\pi ^t$$ and action $$a^t$$ is defined as $$r^t(\pi ^t,a^t)=\sum _{i \in \mathcal {S}}\pi ^t(i)r^t(i,a^t)$$ where $$r^t(i,a^t)$$ denotes the reward accrued in one cycle (3 months) in each health state *i* given action $$a^t$$. The reward is measured in NMB, a measure that captures both health outcomes (measured in quality adjusted life years, or QALYs) and financial costs. NMB is a commonly used metric of value in the health policy literature [38, 58]. QALYs are a measurement of health outcomes, and are a function of both length of life and quality of life, and vary by health state [47]. The NMB is defined as $$\lambda $$(*QALYs*) - *Costs*, where $$\lambda $$, the *willingness-to-pay threshold*, is a weight that translates health improvements to dollar values so as to be comparable with the financial costs. As suggested by previous literature, we use a $$\lambda $$ value of $150,000/QALY gained [44].

If a patient is in CKD stage 3 or above ($s^t = 4,5,...,9$) and has a positive test result, he/she will start treatment, accrue a lump-sum terminal reward, $R^t(s^t)$, and quit the decision process. $R^t(s^t)$ denotes the remaining discounted future NMB of the patient if the decision process terminates at period *t* with health state $s^t$, which considers the remaining lifetime costs and quality of life. Also, we assume that at age 85 (at the final decision epoch *T*), no further action can be taken and the patient also accrues a terminal reward $R^T(s^T)$.

Any decision rule that satisfies Bellman’s equation is an optimal policy [48]. Our objective is to find a series of actions that maximizes our value function *v*, the expected sum of discounted NMB across all decision epochs:

2 $$\begin{aligned} v^{*,t}(\pi ^t) = \max _{{a}\in \mathcal {A}} \{v^t(\pi ^t,{a})\} \end{aligned}$$

In our problem, the value function *v* can be expressed as:

3 $$\begin{aligned} v^t(\pi ^t,a^t)= & {} r^t(\pi ^t,a^t) \nonumber \\{} & {} + \gamma \sum _{i \in \mathcal {S}} \sum _{j \in \mathcal {S}} \sum _{k \in {\textbf{O}}} \pi ^t(i) p^{t}_{ij} q^{{a}}_{jk} v^{*,t+1}(\pi ^{t+1}) \end{aligned}$$

for t = 1,..., T-1, with boundary conditions

4 $$\begin{aligned} v^T(\pi ^T)= \sum _{i \in \mathcal {S}}\pi ^T(i){R}^T(i) \end{aligned}$$

The stage reward, $r^t(\pi ^t,a^t)$, denotes the NMB of one cycle (3 months). The stage costs include the cost of the screening, screening laboratory costs, doctor costs, and clinic costs. We assumed the stage cost of screening occurs immediately for screening, and that there is no stage cost for waiting. The discount factor, $\gamma$, is the 3-month weighing factor (set to be equivalent to 3% discount annually). Note that $p^{t}_{ij}$ is a function of age, gender, race, proteinuria status, and treatment status. The terminal reward, $R^t(s^t)$, denotes the NMB of the remaining lifespan of the patient, which considers the remaining lifetime costs and quality of life. Costs include all the lifetime costs associated with having CKD (checkups, medication, doctors, etc.). The transition probabilities, $p_{ij}^{t}$, stage rewards, $r^t(\pi ^t,a^t)$, and terminal rewards, ${R}^T(i)$, are all risk-factor dependent. Specifically, they vary by proteinuria status, age, race, gender, and treatment status. Therefore the value function takes these risk factors into account, and the POMDP policy can vary by these demographic characteristics.

### POMDP solution process

To identify the preferred action for each feasible belief at each epoch, we use a numerical procedure whereby we reduce the problem to have a discrete set of possible beliefs at each epoch. We do this by choosing a single possible initial belief (e.g., the national prevalence of each health state at age 30 for that demographic group in the U.S., as observed in the NHANES data), which reflects a reasonable initial belief for a physician faced with a new diabetic patient at age 30 potentially needing CKD screening. For every action (*screen* or *wait*) and possible observation outcome, there then exists a particular possible belief for the patient at the next epoch. Given the limited number of actions and observations, as well as the finite number of epochs in this specific problem, this generates a manageable set of possible feasible beliefs for the patient at each epoch across the whole time horizon, with associated probabilities for being at those beliefs (see Appendix A for details). In structure, this problem is then similar to that of a finite-horizon Markov decision process, and we solve it exactly using backward induction using the terminal and stage rewards described above. This solution process has been used in prior POMDP literature [56]. We repeat this solution process for each demographic group of interest.

### POMDP outcomes and approximation of a national policy

To evaluate solution performance, we simulate the POMDP policy using our microsimulation. We use the National Health and Nutrition Examination Survey (NHANES) to estimate the nationally-observed average prevalence of CKD and proteinuria in each demographic group to inform our initial belief in the microsimulation. The simulation then tracks the beliefs of each simulated individual over time, updating them over time as a function of age, testing results, etc. These beliefs determine whether the patient is recommended for testing at the next epoch (and if recommended for testing, a test result is observed). We then report the resultant QALYs, costs, and NMB for each simulated demographic group. We similarly find the outcomes if a status quo screening policy was used instead to allow for comparison.

However, POMDP solutions are belief-specific, and physicians may prefer more general guidelines that are easier to remember and understand. We also want to compare our results with current guidelines, which are given as screening frequencies by age intervals. For instance, the current screening recommendation for those with diagnosed diabetes is to screen every year for all ages. We therefore created a non-belief dependent screening policy by race group and age based on the expected screening frequencies for a US cohort. We simulate individuals’ health states and test results as if they were subject to the POMDP policy, using our transition probabilities and observation probabilities. We can then identify the national average annual screening frequency by age group across race and gender. There are alternative ways of representing this information; in particular, Schell et al. [53] suggests using a statistical approach to estimate screening intervals based on demographic factors. The results we present would be equivalent to this approach if we followed an ordinary least squares (OLS) linear model controlling for race, gender, and 10-year age bins.

**Table 2:** POMDP parameters

Description	Value	Source
Discount rate	0.03 annually	[42]
Epoch length	3 months	Assumed
Initial age	30	Assumed
End age	85	Assumed
Initial belief	See Appx. Sec. D	NHANES
Transition probabilities	See Appx. Sec. D	Microsimulation outputs
Observation probabilities	100%	Assumed
Willingness-to-pay	$150,000/QALY	[44]
Stage rewards	See Tables 9 and 10	Microsimulation inputs
Terminal rewards	See Appx. Sec. B	Microsimulation outputs

## Numerical analysis

In this section, we discuss model parameterization, which draws from nationally-representative data, the medical literature, and a microsimulation model of CKD. We then present the POMDP policy results (including belief trajectories and recommended screening actions for example patients), as well the average impact on each demographic cohort (as evaluated through simulation of the POMDP policy). We contrast the NMB, cost, and QALY outcomes with those in the status quo. We then discuss the approximate national policy, which provides a non-belief-dependent interpretation of the POMDP policy. We then discuss sensitivity analysis outcomes.

### Model inputs

A summary of model input values are given in Table 2. We estimate transition probabilities between health states and terminal rewards using the microsimulation described in Section 4.1.1. We assume perfect sensitivity and specificity for observation probabilities. We use a willingness-to-pay threshold of $150,000 per QALYs gained for the base case analysis. The stage rewards are consistent with the microsimulation inputs. To facilitate the design of practically implementable national guidelines, we additionally provide a non-belief-dependent national policy simulated from the POMDP solution.

#### Microsimulation for POMDP input parameters

Since CKD data by age is scarce, we use a microsimulation to inform the demographically-specific, time-varying transition matrices and the terminal rewards of the POMDP. In our microsimulation, we follow the general structure and inputs of the CKD Health Policy Model to simulate the incidence, progression, and treatment of CKD over time [28]. Our microsimulation simulates the natural history of CKD for individuals from age 30 through death or age 85. This allows us to track disease progression (health states), costs, and QALYs incurred by simulated agents. Critically, the microsimulation accounts for all of the risk factors in our POMDP model (age, race, gender, proteinuria status) as well as treatment, and additionally includes other factors such as cardiovascular health, smoking status, and hypertension which might change CKD risk over time. While the POMDP does not account for these factors directly, as they would overly expand the state space, inclusion in the microsimulation allows us to account for these factors indirectly.

**Table 3:** Summary of screening frequencies by age for four example individuals (one from each demographic group) using the POMDP policy, and age when started treatment (W = White, B=Black; M=Male, F = Female)

	Ages at which an example individual					
	starts screening at least every:					
	2 yrs	1.5 yrs	1 Yr	6 mo.	3 mo.	Trt
WM	34.25	37.5	42.25	52	62.5	67
BM	31.5	36.5	41.25	50	59	75.75
WF	35.25	38.5	44.75	56.25	66.25	78.75
BF	31.5	36.75	41.5	50.5	61	78.75

Patients who receive treatment in the simulation will experience benefits through reduction in the rate of eGFR decline, reduction in proteinuria progression, and a reduction in mortality. We use a 32.7% reduction in annual eGFR decrement in our base case analysis along with a 55% reduction in proteinuria progression. Prior literature indicates that the treatment effect on the rate of eGFR decline can range form 20% to 40% [27], and this is explored in sensitivity analyses. We also use a 23% reduction in mortality as documented in prior medical literature [27].

We validate our outputs with the CKD Health Policy Model. Because the 2015 CKD Health Policy Model was the latest version of the model during our analysis, we also validate our microsimulation outputs (such as cost estimates and life expectancy by CKD stages) with more recent outcomes from other studies. We also updated the input parameters (such as mortality rates) if there was a more current source. Details on microsimulation inputs, outputs, and validation are in Appendix B.

### Numerical results

In the POMDP solution policy, the frequency of recommended screening increases from age 30 to 70, then decreases. This can be explained by the rising risk of CKD with advancing age and the competing effect of declining screening benefit at later ages (lifetime QALYs decrease as the patient’s additional life expectancy declines). We provide the belief and screening outcomes for an example White male in Appendix Table 25. This table provides a summary of the beliefs at each epoch, along with the screening action recommended by the POMDP policy. We observe that the screening frequency weakly increases over time. Due to the length and relative complexity of this table, we have summarized it (along with the analogous results from other demographic groups) in Table 3 for ease of understanding.

**Table 4:** Health benefits from using the POMDP policy over the status quo policy, from simulation output

	Ave. Averted Cases/100,000 pop.		
	Stage 4	Stage 5	ESRD
White Male	1064	3590	3599
Black Male	331	362	771
White Female	787	70	−47
Black Female	114	634	301

Averted cases = (number of cases without the intervention - number of cases with the intervention) per 100,000 population

**Table 5:** Comparison between status quo policy (annual screening among diabetics) and the POMDP policy

	NMB		Costs		QALYs	
	SQ	POMDP	SQ	POMDP	SQ	POMDP
White Male	2,310,857	2,318,699	586,943	587,328	19.319	19.374
Black Male	2,129,187	2,130,896	559,004	558,594	17.921	17.930
White Female	2,470,132	2,472,451	663,189	664,247	20.889	20.911
Black Female	2,437,325	2,439,360	653,966	654,523	20.609	20.626

The table provides average per-person values

Implementation of the POMDP policy generally results in health benefits for the simulated population. In Table 4, we summarize the number of averted cases of CKD stage 4, 5, and end stage renal disease (ESRD) due to using the POMDP policy over the status quo (per 100,000 population, in each demographic group). In general, we see that White males see the largest health benefits in averted cases of more severe CKD. This indicates more frequent screening and earlier diagnosis is generally most beneficial to this group among those compared. This outcome is influenced by the rate of disease progression and treatment efficacy (rapid disease progression even when on treatment means that earlier diagnosis is relatively less able to avert advancement to higher CKD stages). We also observe that the the number of ESRD cases averted among the simulated White female population is very similar to that of the status quo, such that the values are statistically indistinguishable (and the mean number of averted cases is slightly lower), suggesting that ESRD outcomes are the same between the POMDP and status quo policies among White females.

These changes in health outcomes result in differences in lifetime QALYs and costs. The NMB, costs, and QALY values associated with the status quo policy and the POMDP policy estimated from the microsimulation are shown in Table 5. We find that the POMDP policy increases the NMB for all race and gender groups compared to the status quo. Compared to the status quo, using the POMDP policy would increase NMB by $7,842 per patient for White males, $1,709 for Black males, $2,320 for White females, and $2,035 for Black females. Costs in each category are generally higher than the status quo except for Black males. The increases in discounted costs over the remaining lifetime for a 30 year old are $386 for White males, $1,059 for White females, and $556 for Black females, respectively, using the POMDP policy. However, costs are instead $411 lower for Black males. The differences across demographic groups demonstrate the importance of race and gender as screening risk factors, which is also seen in some screening recommendations (see Section 2.2).

Lifetime QALYs are lower for Black individuals compared to their White counterparts in both status quo and POMDP policies, consistent with existing literature [28]. However, the POMDP policy achieves higher discounted QALY values for the remaining lifetime for all groups, with increases of 0.05 for White males, 0.01 for Black males, 0.02 for White females, and 0.02 for Black females respectively. These values are small, and we explore the effect of input uncertainty in our sensitivity analyses (see Section 5). However, they represent meaningful increases in the quality/duration of life, particularly over a large population (see discussion section for population-level estimates of total QALY benefits).

Note that an intervention may avert a large number of cases but still result in modest NMB benefits if the costs are high, the QALY improvement is small, there is limited benefit in life years if the disease is averted, or some combination of these factors. It is therefore worthwhile to report these different measures to provide a more complete picture of what the policy change can achieve. In this case, we find that while the number of averted cases is relatively striking, the NMB benefit is modest. This is likely because more severe stages of CKD generally occur among older adults with limited life expectancy, and preventing transition of CKD stage 3 to higher CKD stages therefore only come with modest QALY increases.

### Policy implications

We follow the procedure described in Section 3.4 to find the approximate national policy. The recommended screening frequency is summarized in Table 6.

The POMDP policy recommends screening slightly less than the status quo policy for ages 30-40, on average. However, it recommends more screening for ages 41-75 for all groups. This difference is particularly stark in ages 51-60 and 71-80, where the POMDP policy generally recommends twice as much screening than the status quo, and ages 61-70, where the POMDP recommends three times as much.

In general, we see that Black individuals should be screened at least as frequently as their White counterparts, if not more frequently, in every age group. The approximate national policy is consistent with the status quo policy in having similar starting and stopping ages for screening, as the POMDP policy recommends screening around age 31 as the earliest age to screen, and 78 as the latest.

**Table 6:** Approximate national policy

	Age at earliest	Age at	Average annual screening frequency				
	screening	last screening	Age 30-40	Age 41-50	Age 51-60	Age 61-70	Age 71-80
White Male	32.25	76	0.79	1.32	2.26	3.62	1.80
Black Male	31.5	75.75	0.83	1.62	2.64	3.62	2.21
White Female	32.75	78.75	0.53	1.10	1.79	2.72	2.01
Black Female	31.75	78.75	0.81	1.48	2.32	3.62	2.01

The average annual screening frequency is the average number of times an individual in that age group would be screened per year, on average. For reference, the status quo policy of screening once annually would result in an average annual screening frequency of 1

## Sensitivity analysis

There exists much variation and uncertainty in some model parameters for CKD, such as in costs and treatment effectiveness, which can vary between patients and health systems, and may sometimes be poorly measured in empirical data. For instance, treatment effectiveness can range from 20% to 40% [26], and there are different methods to estimate CKD medical costs which can lead to different reported values. We therefore perform sensitivity analyses to explore what would happen if the identified POMDP solution (with all its assumptions about input values, sensitivity, and specificity) were implemented in a world where the input parameters varied from our assumed values. We compare these outcomes to a scenario where the status quo policy was implemented instead to understand whether the POMDP policy would be preferred over the status quo policy under these scenarios. To do this, we simulated the POMDP policy and status quo policy using our microsimulation model where the inputs were changed to the values described in the following subsections.

### Treatment effectiveness

In our base case analysis, we assumed that the effect of treatment is a 32.7% reduction in annual eGFR decrements [26]. Prior literature indicates that the treatment effect can range from 20% to 40% [27]. We therefore conducted sensitivity analysis on treatment effectiveness using 20% and 40% reductions to compare with our base case results. To do this, we changed the annual eGFR decrement to either 20% or 40% for all treated individuals in the microsimulation to estimate total discounted lifetime QALYs and costs by demographic group and age of treatment initiation. We then apply the POMDP policy with these eGFR decrements to understand whether the POMDP policy would still outperform the status quo if these were the true CKD treatment effectiveness.

#### Outcomes

When we vary the treatment effectiveness between 20% and 40%, we observe that the QALYs and costs generally increase with higher treatment effectiveness (see Tables 15 and 16). With improved treatment effectiveness, CKD progresses more slowly and patients have longer lifespans, resulting in higher QALYs and costs in both the status quo and POMDP policies. We also observe a reduction in the additional health benefits provided by the POMDP policy compared with the status quo with higher treatment effectiveness. This is expected as more effective treatment can prevent patients from progressing to more severe stages for longer periods of time, thereby reducing the benefits of early treatment initiation. Despite this, the POMDP policy provides higher QALYs and NMB in all treatment effect scenarios except for Black males where treatment has an effectiveness of 40%. For this case, the comparative advantage of the POMDP policy is sufficiently small that the discounted lifetime QALYs and costs garnered in both the POMDP and status quo policies are sufficiently similar to be affected by stochastic noise in the simulation, resulting in a slight negative incremental NMB compared to the status quo policy. We evaluate outcomes using the individual-level simulation, which relies on random sampling to simulate events. This can lead to small variation in simulation outputs even when multiple iterations are averaged together to provide more stable estimates. When the QALY outcomes are sufficiently similar, the differences can be within the range of these small variations. This suggests the benefits of the POMDP policy are subject to uncertainty in treatment effectiveness, particularly for Black males.

### CKD medical costs

Heterogeneous financial health systems in the US have resulted in variation in estimations of CKD medical costs. In our base case, we drew cost estimates from [28]; however, we also consider another widely used set of medical expense values: the United States Renal Data System (USRDS) report. The USRDS report estimates are generally higher than in our base case for CKD stage 1-3, and aggregates costs for stages 4, 5, and ESRD. Specifically, the USRDS reports average costs of $28,913 for CKD stage 1-2, $30,780 for CKD stage 3, and $41,976 for stage 4-5. In comparison, [27] reports the following costs for CKD stages 1 to 4, and stage 5 with ESRD, respectively: $12,505 for CKD 1, $17,049 for CKD 2, $18,263 for CKD 3, $25,048 for CKD 4, and $76,153 for CKD 5 with ESRD. We therefore used the USRDS costs as a sensitivity analysis to evaluate whether these differences in costs can affect the benefits brought by our optimal policy.

Another commonly used reference for CKD cost estimates is Honeycutt et al. [29], which use estimates of $1,600, $1,700, $3,500, and $12,700 per year for CKD 1-4, respectively. We therefore used these costs in a separate sensitivity analysis on costs.

#### Outcomes

The cost estimates provided by the USRDS report are generally higher for stage 0-4 patients, but lower for stage 5 and ESRD patients compared to our base case values. Additionally, the USRDS report cost estimates only stratified by age, whereas our base case costs are stratified by age, gender, hypertension, and proteinuria status. We observe lower lifetime costs for all cohorts when using the USRDS report cost estimates, which is primarily driven by the cost differences in the ESRD cohort (see Table 17). As expected, we find that the QALY outcomes remain the same as the base case scenario; this results in higher NMBs overall. Unlike in the base cases analysis, in this scenario, we find that the POMDP policy is not cost-effective for the Black patient cohort, suggesting the POMDP overestimates the correct number of CKD screenings needed. This difference arises because the USRDS report cost estimates do not consider hypertension and other comorbidities, whereas the POMDP policy accounts for the fact that the Black patient cohort is more affected by hypertension and other comorbidities.

In comparison to our base case analysis, the Honeycutt costs provide significantly lower estimates for stage 0-4 patients. Since the cost estimates from Honeycutt do not include stage 5 and ESRD costs, we use the costs from our base case analysis for these CKD stages. As expected, we again observe that the QALYs remain the same, and the NMB increases for all cohorts. We observe a substantial disparity in costs between the Black and White patient cohorts, with decreased costs for the White patient cohort increased costs for the Black patient cohort when using these lower cost estimates (see Table 18). We attribute this to the higher number of patients in stage 4, stage 5, and ESRD within the Black patient cohort – the increased costs for the Black patient cohort, compared to the base case, is primarily driven by Black patients entering ESRD earlier.

Overall, these sensitivity analyses on costs indicates that the large uncertainty in the true costs of medical care, which can include comorbidities affected by CKD status, can substantially affect POMDP policy performance. While the base case cost values are the most reasonable costs to use to evaluate a national level policy, these outcomes suggest that regional cost estimates should be used if the policy is to be adopted locally.

### Disease progression

Our CKD progression depended on eGFR decrement by age used in the microsimulation (see Appendix Table 7), which used estimates from [28]. However, there is some evidence that these values are underestimates of CKD progression probabilities [28, 64]. We therefore increased the decrements in Table 7 until microsimulation outputs for CKD prevalence projections are at the upper 95% confidence bound provided by [28] using NHANES data (this ensures the increase in progression rates is still reasonable). Through this process, we found that we can increase eGFR decrements by 10%. We then repeated our analysis and identify whether the POMDP policy would still outperform the status quo after implementing this change.

#### Outcomes

When we increase the annual eGFR decrements in Table 7 by 10% and rerun the analysis using the original optimal policy, we find that the NMB, costs, and QALYs decrease for all cohorts compared to the base case analysis (see Table 19). This is expected, because with faster disease progression patients will progress to higher CKD stages more rapidly and experience higher mortality rates more quickly compared to the base case analysis. In general, the POMDP policy generates relatively fewer QALYs compared to the status quo policy, as accelerated disease progression continues after treatment is initiated (making earlier diagnosis less beneficial).

The POMDP policy remains cost-effective compared to the status quo for all groups besides Black males, who had the smallest QALY gain in the POMDP policy relative to the status quo even in the base case. The disease progression rate for Black males is the highest among all demographic groups, so they are the most affected by the accelerated progression rate. The QALYs in the POMDP policy and status quo become sufficiently similar that variation due to the sampling processes in the simulation becomes a factor, resulting in lower QALYs in the POMDP policy and negative incremental net monetary benefits.

### Willingness-to-pay (WTP) threshold

We used a willingness-to-pay threshold of $150,000/QALY gained in our base case analysis. However, there is controversy about what the appropriate WTP should be [34, 54], and we therefore also evaluated the POMDP policy outcomes using WTP thresholds of $50,000/QALY gained and $100,000/QALY gained.

#### Outcomes

As expected, when using a WTP threshold of $100,000/ QALY gained or $50,000/QALY gained, we observe a decrease in the NMB from the base case outcomes (which use a threshold of$150,000/QALY gained). However, the POMDP policy remains cost-effective for all race and gender groups across all WTP thresholds (see Tables 20 and 21). This indicates the POMDP policy is not sensitive to the choice of WTP thresholds even if health benefits are valued relatively less per dollar, over a reasonable range.

### QALY weights

We drew our CKD stage-specific QALY weights from [12], as this is one of the most widely used estimates in recent literature. Our proteinuria QALY weights were sourced from [27]. However, our base case QALY outcomes neglected the quality-of-life effects of comorbidities (heart disease, stroke, etc.). We therefore included QALY weights from [27] for these conditions in sensitivity scenarios to evaluate the impact on health outcomes.

#### Outcomes

We observe higher NMB and QALYs for all cohorts after using the QALY weights used in [28] (see Table 22). We expected higher QALYs and NMB values as these QALY weights assign smaller decrements as CKD progresses to the next stage compared to the base case, resulting in higher QALYs for CKD stages. The POMDP policy remains cost-effective benefits for all cohorts when compared to the status quo policy, indicating it is not sensitive to these choices of QALY weights.

### Imperfect test sensitivity and specificity

Our base case model assumes the CKD diagnostic test is perfect, as diabetic individuals are likely to have multiple confirmatory follow up tests after a positive result (thus unlikely to have a false positive) and several tests if a physician observes symptoms of CKD (unlikely to have a false negative). Consistent with this, the microsimulation assumes a screened individual will undergo two proteinuria tests, and if at least one is positive, the patient’s eGFR level will be measured. In the simulation, all eGFR levels detected below 60 will be placed on treatment. In the base case, we assume 100% sensitivity and specificity for both proteinuria and eGFR measurements.

However, there may be other factors (irregular contact between patients and medical providers, etc.) that may lead to a single test result determining the outcome for a patient within an epoch, and we therefore perform sensitivity analysis around test sensitivity and specificity. For model clarity and simplicity, we adjust the test sensitivity and specificity for the proteinuria test (sensitivity of 0.76 and specificity of 0.96 [26]) and use the same testing algorithm as described above for each CKD screening. As with the other sensitivity analyses, we apply the POMDP policy to a simulation that uses these values for the diagnosis test characteristics to observe the impact on QALY, cost, and NMB outcomes of the POMDP policy compared to the status quo policy.

#### Outcomes

When we incorporate imperfect test characteristics for the simulated diagnostic test, we observe similar QALYs, costs, and NMB (see Table 23). The POMDP policy remains cost-effective for all demographic groups compared to the status quo. This suggests that the POMDP policy is likely to be beneficial even if the diagnostic test is performed once, without follow up (which is unlikely in current standard practice).

### Simultaneous evaluation of uncertainty scenarios

Variation in the above parameters may lead to different outcomes if they are evaluated alone or simultaneously. We therefore additionally included a scenario where we varied all of these parameters simultaneously (besides WTP) across the values described above (we fix WTP to be $150,000/QALY gained for interpretability). To do this, we assume there is equal probability of realizing each of the value levels described above for each parameter. We simulate the life trajectories for individuals from age 30 to age 85 for each race group while either using the status quo policy or the POMDP policy. We can then compare NMBs, costs, and QALYs between policies. We repeat this process up to 72 times separately for each race and gender group and report the average outcomes.

#### Outcomes

The average simulated NMB, cost, and QALY values are summarized in Appendix Table 24. Outcomes for all demographic groups besides Black males show higher NMB, higher costs, and higher QALYs for the POMDP policy compared to the status quo. The 95% confidence intervals for the QALYs and costs for the Black male cohort overlap between the POMDP and status quo policies, indicating that differences are not statistically distinguishable at the 5% significance level; while the average QALYs, costs, and NMB are slightly smaller in the POMDP policy compared to the status quo, these differences are within the range of variation due to the sampling processes in the simulation. This may be due to some of the effects discussed in the one-way sensitivity analyses, where we also saw effect sizes within the noise for Black males. In general, these outcomes indicate that although the POMDP policy is sensitive to input parameters, for all cohorts besides Black males there may be reason to believe that the status quo does not screen for CKD frequently enough and the POMDP policy would be an improvement.

## Conclusions

Current screening guidelines for CKD do not simultaneously consider personalized information on past test results, race, gender, proteinuria status, and age. In this work, we identify a policy that considers these factors for patient-specific screening to improve patient health.

However, national guidelines typically do not base their screening recommendations on belief states. To approximate this POMDP national policy in terms of more typical recommendation criteria (age, race, and gender), we conduct a simulation analysis to identify a fixed age interval, fixed frequency screening policy. We find that the POMDP policy suggests more frequent screening after age 40 for all race and gender groups compared to status quo policy. Based on the NHANES data, there are roughly 43 million individuals at age 30-39 in the U.S., of whom 3% are diabetic. Of this population, 23.65%, 22.17%, 30.54%, 22.17%, are White males, Black males, White females, and Black females respectively. If our POMDP policy is used, we would expect to gain 33,018 additional QALYs and $4.3 billion more in total NMB, for a additional cost of $576 million. To provide some context for this cost, note that Medicare fee-for-service spent $85.4 billion in 2020 on beneficiaries with CKD [60].

We also quantify the health outcomes and identify the POMDP policies associated with the scenarios explored in our sensitivity analyses. We examine several sensitivity analyses to examine the effect of variation in costs, WTP thresholds, QALY scores, treatment effectiveness, disease progression by age, race, and gender. We find that the POMDP policy is sensitive to uncertain parameters, particularly medical costs and disease progression rates.

There are several limitations to this work. While we draw our input values from empirical data and recent medical literature, uncertainty remains in our input parameters. In addition, we used uniform distributions when considering simultaneous variation in inputs; in reality, this is unlikely to be the case. However, given the scarcity of data on the correct distribution of uncertain model parameters, we use naive, uninformed uniform distributions in our sensitivity analyses. For our estimation of rewards, we did not use race- and gender-dependent QALY weights, nor did we include non-medical financial costs (e.g., caretaker costs, etc.). However, note that the rewards still vary by race and gender since we used demographic-specific costs and adopted race/sex-specific transition matrices to estimate the expected future rewards from the microsimulation. Data on race- and gender-specific QALY values and costs and patient costs for CKD are sparse. This calls for further measurement of patient-specific CKD treatment and screening costs for future work. This study also only considers a small subset of the risk factors that influence CKD progression and development since we focus on people who have diagnosed diabetes and unknown proteinuria. Not including other risk factors may reduce the accuracy of our POMDP policy.

Despite these limitations, we believe this study sheds light on the POMDP CKD screening policy in the U.S. Our research highlights the importance of early risk detection on CKD through the presence of time-varying risk factors. Screening for CKD in high-risk populations is cost-effective and presents a critical opportunity to detect CKD in early stages with the intent of identifying and managing CKD and diagnosing the etiology of CKD. Our results also suggest that CKD screening policy should vary by race and gender. Our study is the first to provide race and gender-specific screening recommendations using a POMDP framework. This recommendation will hopefully lead to smaller disparities in care and disease outcomes. Better forecasts of the future burden of CKD can help policy makers prepare for future health care needs and raise awareness among patients and clinicians about the importance of CKD screening.

There is much future work remaining to improve CKD screening guidelines for diabetic patients. Patients may face barriers to adhering to screening guidelines, and such challenges may also vary by population subgroup, resulting in disparities in diagnosis and care. More work also needs to be done on individually-tailored treatment regimens, as CKD progression rates and ability to adhere to treatment may vary across the population. Quantitative modeling frameworks like the one used here may provide a useful tool in addressing these challenges.

## Figures and Tables

**Fig. 2:**
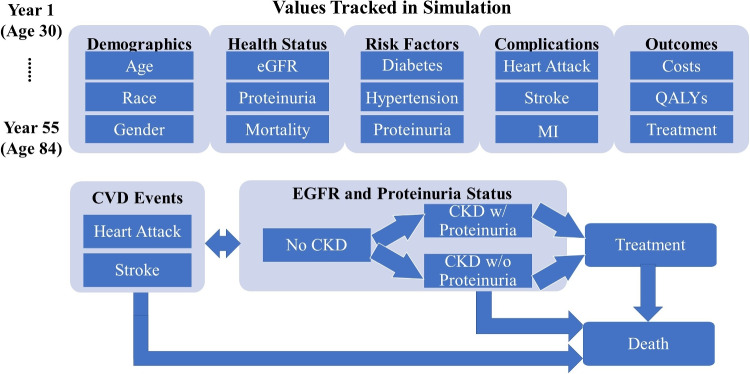
Key components of the microsimulation model

**Fig. 3:**
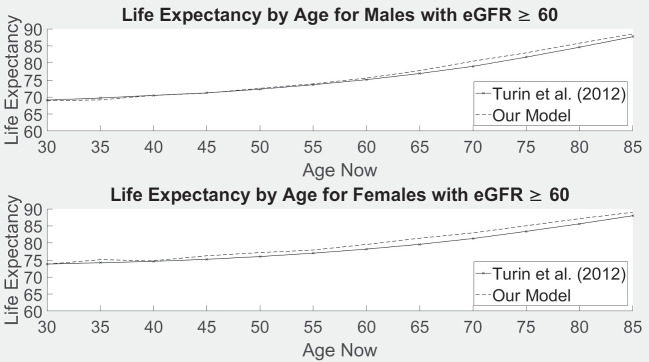
Life expectancy by current age for males and females with eGFR $\ge$ 60

## Data Availability

This work used only data from published literature and publicly available sources.

## Appendix A: POMDP solution methodology

We here describe the POMDP solution methodology used in this study. This POMDP solution method was chosen because it was easy to numerically implement, provided an exact solution, and solved the problem at hand in a reasonable amount of time on available computational resources.

We first identify the set of beliefs used in the entire time horizon with associated stage and future rewards, then use backward induction to solve for optimal actions at each belief. To identify all possible beliefs used in the time horizon, we first consider the set of N beliefs for a patient at time *t* for a particular cohort (e.g., white males). Over the next time epoch, he may transition to an unobserved health state, which we capture by multiplying the beliefs by the transition matrix. Then there are three action/observation outcomes. We either observe that he is dead (belief becomes [0 0 0 0 0 0 0 0 0 1]), he is alive and not screened (N possible beliefs as the result of the original beliefs times the transition matrix), or he is alive and screened. The perfect screening test allows the health state to be observed, resulting in 9 possible beliefs, each with all zero elements besides one 1 value, corresponding to the 9 non-dead health states (100% belief in a particular health state). Of these, patients observed to have CKD stage 3 and above initiate treatment and decisions terminate, so only 3 beliefs ([1000000000],[0100000000], and [0010000000]) need to be considered at the next epoch. Thus there are a total of N + 4 beliefs at the next epoch. This repeats, with linear growth in the number of beliefs over time.

This then becomes a very tractable number of beliefs given our particular problem, as we start with one belief at age 30 (the nationally representative prevalance for that cohort, as estimated using NHANES data). For example, for a white male, this belief vector is [0.75 0.2292 0.0208 0 0 0 0 0 0 0]. The belief set at the next epoch is [1000000000], [0100000000], [0010000000], [0000000001], and [0.7474 0.2270 0.0242 0.0005 0.0009 0 0 0 0 0]. These beliefs correspond to observations of test outcomes of healthy, stage 1 CKD, stage 2 CKD, dead, and alive with no screening outcome, respectively. This process then repeats for the next decision period. By the end of our time horizon, there are only 881 beliefs at the last period and only a total of 96,580 unique beliefs that we need to consider in computer memory.

Each belief is then associated with a reward (in NMB). We use backward induction to find the expected future rewards associated with the actions at a particular belief for each epoch. We then use these to identify the optimal actions (the action that generates the larger expected future reward).

## Appendix B: Microsimulation model description

We closely followed the microsimulation presented in [28] to simulate the natural course of CKD among diabetics (see Appendix Fig. 2). Specifically, we used the same state space and eGFR decrements, along with the risk of other risk factors (CVD, hypertension, and smoking), as well as the same relative risk of mortality. We also used the same cost values (converted to 2020 dollar values) and QALY values for our base case analysis. We then updated model inputs for disease prevalence and death probabilities by following the approach in [28] using the latest NHANES input parameters.

We give a brief overview of the microsimulation states and structure here. We used the same CKD states as [28]. CKD is defined as abnormalities of kidney function for more than three months, and proteinuria is a general term for the presence of increased amounts of protein in the urine. We followed the National Kidney Foundation-Kidney Disease Outcomes Quality Initiative (NKF-KDOQI) guidelines to define the CKD stages by estimated glomerular filtration rates (eGFRs) and the presence of kidney damage (see Table 1) [13]. We defined kidney damage (proteinuria) as presence of microalbuminuria or macroalbuminuria depending on the levels of albumin in the urine [26, 28].

As in [28], we assumed individuals with CKD do not recover and the death state is absorbent. Most kidney diseases do not have symptoms until later in their course and are detected only when they are chronic. Most causes of CKD are irreversible, and treatment is aimed at slowing progression to kidney failure.

In the microsimulation model, a patient with diabetes is assigned an initial eGFR and proteinuria status based on the eGFR distribution and proteinuria prevalence estimated from NHANES data and then experiences annual decrements in eGFR and proteinuria incidence based on current eGFR level, age intervals, race, gender, and the presence of proteinuria where individuals without proteinuria may stochastically acquire proteinuria each period [28]. Proteinuria progression rates are also estimated from NHANES data. eGFR and proteinuria status are the key components for tracking progression of CKD stages. We assume eGFR only declines deterministically over time given current eGFR value, age interval, race, and proteinuria status. The eGFR decrements used in the microsimulation are summarized in Table 7, which is modified from [28].

**Table 7:** Annual eGFR decrement by age

Status	eGFR	Age 30-49 y		Age $\ge$ 50 y	
		White	Black	White	Black
No proteinuria	$\ge$60	0.935		1.1	
	$30-59$	2.45	3.5	2.45	3.5
	< 30	2.8	4.2	2.8	4.2
Proteinuria	$\ge$60	4.1			
	$30-59$	4.55	6.5	4.55	6.5
	< 30	5.2	7.8	5.2	7.8

**Table 8:** Relative risk of mortality by eGFR thresholds

Description	Value	Source
Relative risk of mortality		
eGFR $\ge 60$	1	[27]
eGFR $45-59$	1.1	[27]
eGFR 30-44	1.7	[27]
eGFR 15-29	3.4	[27]

Death was assigned through an annual probability based on an adjusted all-cause mortality rate that accounts for baseline, cardiovascular disease (CVD), and CKD mortality rates reflecting the increased risk from CKD (see Table 8), CVD mortality rates determined by myocardial infarctions (MI), stroke events, and ESRD mortality rates. Risk factors and medical events are simulated annually using probability functions. Individual-level risk factors and events are simulated for all CKD stages except ESRD, which is modeled by assigning mean population cost, mortality, and utility values derived primarily from USRDS data. We follow the assumptions of [26] that patients who survive 1 year in stage 5 will reach ESRD and require either dialysis therapy or a transplant [26]. This allows us to combine CKD stage 5 and ESRD together.

**Table 9:** QALY values

Description	Value	Source
Baseline	1.00	[27]
Death	0.00	Assumed
QALY decrements		
Proteinuria	0.01	[12]
eGFR $60-90$ with Proteinuria	0.15	[12]
eGFR $30-59$	0.20	[12]
eGFR $15-29$	0.26	[12]
eGFR < 15	0.27	[12]

**Table 10:** Annual costs, by patient and treatment usage Characteristics

Description	Value	Source
CKD stage 5	$76,153	[27]
Base expenditures (Excl. Stage 5)	$5,790	[43]
eGFR $$15-29$$	$13,689	[27]
eGFR $$30-59$$	$7,342	[27]
eGFR $$60-89$$	$5,512	[27]
Age	$46 $$\times $$ age	[27]
Female gender	$264	[27]
Proteinuria	$6,165	[27]
Type 2 diabetes	$2,334	[27]
Type 2 diabetes & eGFR 15-29	$5,119	[27]
Screening fee	$182	[27]
Specialist follow-up if eGFR<60	$108	[27]
CKD drug therapy (for patients with T2D)	$607	[27]

The QALY values and incremental costs in Tables 9 and 10 respectively allow us to calculate the NMB. These values are from previous literature and the costs are evaluated from the health care system perspective. We assume that diagnosed CKD patients receive treatment and stop screening. To find the long-run expected NMB after receiving treatment, we included a reduction term in the annual eGFR decrement equation. We found the expected lifetime duration in each state starting at each age, from which we apply cost and QALY values to calculate the expected discounted lifetime NMB.

We simulated 10,000 individuals for each race and gender group to acquire CKD progression data including each individual’s health state, accumulated cost, and QALYs. Microsimulation validation is in Appendix Section C.

## Appendix C: Microsimulation validation

While we closely followed an existing, peer-reviewed simulation of CKD to inform our model structure and transitions, many of our model parameters have been updated to reflect the most recent information. We therefore validated our simulation to ensure that outcomes are realistic.

**Table 11:** CKD lifetime incidence rate at ages 30-49

Stage	Hoerger et al. [28]	Our model
All CKD	54.13 $(53.40-54.93)$	$54.59-55.27$
CKD stage 3+	47.06 $(46.54-47.68)$	$47.52-47.95$
CKD stage 4+	9.42 $(9.02-9.80)$	$9.78-9.85$
CKD stage 5+	3.17 $(2.95-3.37)$	$3.24-3.28$

We began with CKD progression rates. We validated that the resultant model was identical to that in [28] by comparing our model outputs to the original simulation on CKD lifetime incidence rates at age intervals 30-49 and 50-65 (see Tables 11 and 12). We provided two sets of output values where the same input parameters as the [28] study are used (CKD mortality rates and proteinuria prevalence) for the first one (denoted as “Our Model (2015 inputs)”). We also provided the output values where the updated CKD mortality rates (CDC WONDER 2018) and proteinuria prevalence (NHANES 2018) are adopted (denoted as “Our Model (2018 inputs)”) [20]. Our values overlapped with their 95% confidence intervals. The incidence rates for all CKD are higher if the latest inputs are used which is consistent with the projection in [28].

**Table 12:** CKD lifetime incidence rate at ages 50-65

Stage	Hoerger et al. [28]	Our Model
All CKD	52.01 (50.79-53.24)	47.1-54.42
CKD stage 3+	44.7 (43.65-45.74)	41.53-47.45
CKD stage 4+	9.1 (8.63-9.61)	8.19-9.76
CKD stage 5+	3.31 (3.00-3.53)	2.45-3.22

To validate mortality outcomes, we compared life expectancy values by age, gender, and eGFR values from [59] with our model outcomes (see Fig. 3 and [59]). Our values are generally close to their values, but small differences are expected since Turin et al. (2012) focuses on a different cohort (a Canadian cohort in 2008 instead of an American cohort in 2018).

To validate the values used in our terminal rewards, we compared our estimates with values in the medical literature [29]. Estimating the medical costs of earlier stages of CKD is challenging because these stages often go unreported on health care claims or in survey data [16]. Our CKD stage 1 to stage 4 medical cost estimates ($12,505-$25,048) are within the 95% confidence intervals ($4,566-$23,187) provided by [29] (updated to 2020 dollars).

## Appendix D: Estimating an initial belief and transition probabilities for the POMDP

To focus our numerical results on policies pertinent to the US cohort, we started with an initial belief in our POMDP that reflects the prevalence of CKD stages 0-3 within diabetics from age 25-45 in each demographic group as recorded in the NHANES data (waves 2010-2018), see Table 13. There was only one diabetic in stage 4 or above in each demographic group, so they were omitted due to the small sample size. CKD prevalence depends on eGFR and proteinuria. To find this prevalence, we therefore used the following procedure. To estimate eGFR, the CKD-EPI equation is suggested by the NHANES tutorial and the CDC Chronic Kidney Disease (CKD) Surveillance System [15]. We used the CKD-EPI equation to convert creatinine levels to eGFR: 141 x [min(calibrated serum creatinine in mg/dL) /$\kappa$, 1)]^α^ x [max(calibrated serum creatinine in mg/dL/$\kappa$, 1)]^κ^ x 0.993^age^ x (1.018 if female) x (1.159 if Black) where $\kappa$ = 0.7 if female, and 0.9 if male and $\alpha$ = -0.329 if female, and -0.411 if male. We then use the existence of urinary albumin-to-creatinine ratios of 30-299 mg/g (microalbuminuria) and >300 mg/g (macroalbuminuria) to define proteinuria. We apply these equations to individuals in NHANES data, while considering sample weights, to estimate CKD prevalence by age, race, and gender of the main manuscript).

**Table 13:** Initial prevalence of CKD, (ages 25-45, estimated from NHANES data)

Demographic Cohort	Healthy (in %)	CKD1 (in %)	CKD2 (in %)	CKD3 (in %)
White Males	94.76	5.05	0.00	0.19
Black Males	88.28	10.62	1.10	0.00
White Females	89.43	7.71	2.42	0.22
Black Females	85.14	13.77	1.09	0.000

We next turned to estimating transition probabilities for the POMDP. The microsimulation allows us to track health states (no proteinuria/CKD, CKD stage 1-5, death) over time. We run the microsimulation with 10,000 nationally representative samples to track each individual’s heath state from age 30 till death and terminates at age 85. The outputs from the microsimulation model enable us to construct the transition probability matrix by fitting multinomial logistic regressions. We used the current health state, age, race, and gender to estimate the likelihood an individual is in each health state annually. We then use the eigendecomposition numerical approximation methods proposed by [10] to estimate three-month transition matrices from the annual data by raising them to the 1/4 power [10]. For the disease progression for treated individuals, we applied eGFR decrements reduction on the samples and repeat the above steps.

Our microsimulation provides us with annual CKD health dynamics. We estimated the annual transition probability matrix by fitting multinomial logistic regressions to the annual simulation output data, controlling for age, race, gender, proteinuria and treatment status. We further used an eigen-decomposition approach to estimate a 3-month transition matrix from the above annual output values [10]. This allows us to generate different transition probability matrices for each age, race, and gender groups, as well as each proteinuria status (with and without proteinuria) and CKD treatment status (treated or untreated). Validation of transition probabilities is in Appendix Section E.

## Appendix E: POMDP input validation

Our POMDP used transition matrices and terminal rewards estimated from the microsimulation outcomes. To ensure these matrices accurately capture disease progression rates, we used several processes to check these inputs. We first verified that our estimated transition matrices are consistent with the progression rates observed in the microsimulation model (internal validation). We began by setting the initial distribution of CKD prevalence to be consistent with the national estimates for 30 year old in the U.S. We then applied our transition matrices for 35 years to project the prevalence for age $\ge$ 65 (see Table 14).

We used the same inputs as [28] as this prior work validates their annual eGFR decrements with NHANES 1999-2010 data. We also provided the projection if the latest CKD mortality rates and proteinuria prevalence are used. We focused on validating the transition probabilities to ensure our model inputs were consistent with observed values. In general, these results are consistent with the values in the data, increasing our confidence that the transition probabilities reflect observed phenomena.

**Table 14:** Projected prevalence of CKD (% of cohort)

Age $\ge$ 65 y	NHANES	Our POMDP
No CKD	60.35 $(59.13-61.57)$	59.60
CKD Stage 1	1.16 $(0.86-1.45)$	1.48
CKD Stage 2	8.43 $(7.65-9.22)$	8.08
CKD Stage 3	27.47 $(25.68-29.26)$	27.66
CKD Stage 4	2.59 $(2.17-3.01)$	2.41
CKD Stage 5	NA	0.78

## Appendix F: Sensitivity analysis outcomes tables

**Table 15:** Comparison between status quo and POMDP policies given 20% reduction in eGFR progression due to treatment

	NMB		Costs		QALYs	
	SQ	POMDP	SQ	POMDP	SQ	POMDP
White Male	2,292,610	2,299,202	578,644	579,617	19.142	19.192
Black Male	2,110,087	2,115,174	553,986	555,371	17.76	17.804
White Female	2,446,704	2,450,038	652,927	654,236	20.664	20.695
Black Female	2,417,669	2,417,890	643,206	645,031	20.406	20.419

**Table 16:** Comparison between status quo and POMDP policies given 40% reduction in eGFR progression due to treatment

	NMB		Costs		QALYs	
	SQ	POMDP	SQ	POMDP	SQ	POMDP
White Male	2,324,758	2,329,014	592,246	591,522	19.447	19.470
Black Male	2,139,005	2,138,553	560,046	557,487	17.994	17.974
White Female	2,488,700	2,490,267	671,285	673,697	21.067	21.093
Black Female	2,451,110	2,452,647	658,060	659,674	20.728	20.749

**Table 17:** Comparison between status quo and POMDP policies using cost estimates from the USRDS report

	NMB		Costs		QALYs	
	SQ	POMDP	SQ	POMDP	SQ	POMDP
White Male	2,489,833	2,495,879	407,967	410,148	19.319	19.374
Black Male	2,261,754	2,259,122	426,436	430,367	17.921	17.930
White Female	2,689,476	2,691,940	443,844	444,758	20.889	20.911
Black Female	2,618,751	2,615,661	472,540	478,221	20.609	20.626

**Table 18:** Comparison between status quo and POMDP policies using costs from [29]

	NMB		Costs		QALYs	
	SQ	POMDP	SQ	POMDP	SQ	POMDP
White Male	2,867,259	2,877,144	30,541	28,883	19.319	19.374
Black Male	2,617,767	2,620,010	70,423	69,479	17.921	17.930
White Female	3,096,878	3,100,259	36,442	36,439	20.889	20.911
Black Female	3,019,928	3,023,344	71,364	70,538	20.609	20.626

**Table 19:** Comparison between status quo and POMDP policies after enlarging eGFR decrements by 10%

	NMB		Costs		QALYs	
	SQ	POMDP	SQ	POMDP	SQ	POMDP
White Male	2,267,236	2,271,440	581,215	581,229	18.99	19.018
Black Male	2,091,070	2,085,958	557,436	553,330	17.657	17.595
White Female	2,416,508	2,420,687	655,667	657,080	20.481	20.518
Black Female	2,383,881	2,386,811	646,897	646,639	20.205	20.223

**Table 20:** Comparison between status quo and POMDP policies using a $50,000/QALY WTP threshold

	NMB		Costs		QALYs	
	SQ	POMDP	SQ	POMDP	SQ	POMDP
White Male	378,991	381,347	586,943	587,328	19.319	19.374
Black Male	337,059	337,903	559,004	558,594	17.921	17.930
White Female	381,251	381,319	663,189	664,247	20.889	20.911
Black Female	376,464	376,771	653,966	654,523	20.609	20.626

**Table 21:** Comparison between status quo and POMDP policies using a $100,000/QALY WTP threshold

	NMB		Costs		QALYs	
	SQ	POMDP	SQ	POMDP	SQ	POMDP
White Male	1,344,924	1,350,023	586,943	587,328	19.319	19.374
Black Male	1,233,123	1,234,399	559,004	558,594	17.921	17.930
White Female	1,425,692	1,426,885	663,189	664,247	20.889	20.911
Black Female	1,406,894	1,408,065	653,966	654,523	20.609	20.626

**Table 22:** Comparison between status quo and POMDP policies using QALY weights from [28]

	NMB		Costs		QALYs	
	SQ	POMDP	SQ	POMDP	SQ	POMDP
White Male	2,445,457	2,453,175	586,943	587,328	20.22	20.27
Black Male	2,270,277	2,270,977	559,004	558,594	18.86	18.860
White Female	2,662,895	2,665,032	663,189	664,247	22.17	22.20
Black Female	2,611,334	2,613,780	653,966	654,523	21.77	21.79

**Table 23:** Comparison between status quo and POMDP policies using an imperfect test (sensitivity of 0.76, specificity of 0.96)

	NMB		Costs		QALYs	
	SQ	POMDP	SQ	POMDP	SQ	POMDP
White Male	2,310,858	2,318,704	586,941	587,323	19.319	19.374
Black Male	2,129,188	2,130,904	559,003	558,585	17.921	17.930
White Female	2,470,133	2,472,454	663,187	664,244	20.889	20.911
Black Female	2,437,326	2,439,365	653,965	654,517	20.609	20.626

**Table 24:** Comparison between status quo and POMDP policies with simultaneous variation in uncertain inputs

	NMB		Costs		QALYs	
	SQ	POMDP	SQ	POMDP	SQ	POMDP
White Male	2,598,044	2,603,548	341,251	342,263	19.595	19.639
Black Male	2,379,957	2,378,501	353,316	353,228	18.222	18.213
White Female	2,811,600	2,815,142	383,843	383,994	21.303	21.328
Black Female	2,742,747	2,745,396	400,408	402,938	20.954	20.989

**Table 25:** Example white male belief trajectories over time, and screening recommendations from the POMDP policy

Age	Pr(CKD 0-2)	Pr(CKD 3)	Pr(CKD 3+)	Pr(Dead)	Screen
30.25	1.000000	0.000000	0.000000	0.000000	0
30.5	0.747363	0.226964	0.024243	0.000539	0
30.75	0.744735	0.224749	0.027586	0.001079	0
31	0.742116	0.222522	0.030863	0.001618	0
31.25	0.739506	0.220285	0.034075	0.002158	0
31.5	0.736905	0.218037	0.037223	0.002698	0
31.75	0.734312	0.215779	0.040306	0.003238	0
32	0.731727	0.213513	0.043326	0.003779	0
32.25	0.729149	0.211239	0.046282	0.004320	1
32.5	0.726579	0.208958	0.049176	0.004862	0
32.75	1.000000	0.000000	0.000000	0.000000	0
33	0.996387	0.001383	0.000506	0.000849	0
33.25	0.992781	0.002716	0.001026	0.001698	0
33.5	0.989180	0.004002	0.001558	0.002547	0
33.75	0.985585	0.005241	0.002102	0.003396	0
34	0.981994	0.006433	0.002656	0.004244	0
34.25	0.978408	0.007580	0.003221	0.005093	1
34.5	0.974825	0.008682	0.003795	0.005942	0
34.75	1.000000	0.000000	0.000000	0.000000	0
35	0.996299	0.001215	0.000517	0.000970	0
35.25	0.992601	0.002384	0.001045	0.001940	0
35.5	0.988904	0.003508	0.001583	0.002909	0
35.75	0.985209	0.004589	0.002131	0.003877	0
36	0.981515	0.005627	0.002688	0.004845	1
36.25	0.977822	0.006622	0.003252	0.005812	0
36.5	1.000000	0.000000	0.000000	0.000000	0
36.75	0.996188	0.001076	0.000525	0.001090	0
37	0.992374	0.002108	0.001058	0.002179	0
37.25	0.988560	0.003099	0.001600	0.003266	0
37.5	0.984744	0.004049	0.002150	0.004352	1
37.75	0.980926	0.004958	0.002706	0.005438	0
38	1.000000	0.000000	0.000000	0.000000	0
38.25	0.996065	0.000961	0.000530	0.001204	0
38.5	0.992126	0.001882	0.001067	0.002406	0
38.75	0.988183	0.002763	0.001610	0.003607	1
39	0.984237	0.003606	0.002160	0.004806	0
39.25	1.000000	0.000000	0.000000	0.000000	0
39.5	0.995942	0.000869	0.000533	0.001308	0
39.75	0.991879	0.001699	0.001072	0.002614	0
40	0.987810	0.002492	0.001615	0.003917	1
40.25	0.983735	0.003248	0.002163	0.005218	0
40.5	1.000000	0.000000	0.000000	0.000000	0
40.75	0.995800	0.000779	0.000535	0.001421	0
41	0.991593	0.001521	0.001074	0.002840	0
41.25	0.987378	0.002228	0.001616	0.004254	1
41.5	0.983156	0.002899	0.002160	0.005666	0
41.75	1.000000	0.000000	0.000000	0.000000	0
42	0.995637	0.000691	0.000536	0.001544	0
42.25	0.991266	0.001347	0.001073	0.003084	1
42.5	0.986886	0.001969	0.001613	0.004620	0
42.75	1.000000	0.000000	0.000000	0.000000	0
43	0.995492	0.000621	0.000535	0.001650	0
43.25	0.990974	0.001209	0.001071	0.003295	1
43.5	0.986446	0.001765	0.001607	0.004935	0
43.75	1.000000	0.000000	0.000000	0.000000	0
44	0.995332	0.000553	0.000534	0.001763	0
44.25	0.990653	0.001074	0.001066	0.003520	1
44.5	0.985963	0.001563	0.001597	0.005271	0
44.75	1.000000	0.000000	0.000000	0.000000	0
45	0.995156	0.000485	0.000531	0.001884	0
45.25	0.990302	0.000939	0.001059	0.003760	1
45.5	0.985435	0.001363	0.001585	0.005629	0
45.75	1.000000	0.000000	0.000000	0.000000	0
46	0.994965	0.000417	0.000527	0.002013	1
46.25	0.989918	0.000805	0.001050	0.004017	0
46.5	1.000000	0.000000	0.000000	0.000000	0
46.75	0.994810	0.000366	0.000524	0.002115	1
47	0.989609	0.000705	0.001041	0.004220	0
47.25	1.000000	0.000000	0.000000	0.000000	0
47.5	0.994646	0.000315	0.000519	0.002223	1
47.75	0.989280	0.000604	0.001031	0.004434	0
48	1.000000	0.000000	0.000000	0.000000	0
48.25	0.994471	0.000265	0.000514	0.002336	1
48.5	0.988931	0.000504	0.001019	0.004659	0
48.75	1.000000	0.000000	0.000000	0.000000	0
49	0.994286	0.000214	0.000508	0.002455	1
49.25	0.988561	0.000403	0.001006	0.004895	0
49.5	1.000000	0.000000	0.000000	0.000000	0
49.75	0.994089	0.000162	0.000501	0.002580	1
50	0.988168	0.000302	0.000990	0.005142	0
50.25	1.000000	0.000000	0.000000	0.000000	0
50.5	0.993880	0.000111	0.000493	0.002711	1
50.75	0.987752	0.000200	0.000973	0.005403	0
51	1.000000	0.000000	0.000000	0.000000	0
51.25	0.993660	0.000059	0.000484	0.002848	1
51.5	0.987312	0.000098	0.000953	0.005676	0
51.75	1.000000	0.000000	0.000000	0.000000	0
52	0.993426	0.000006	0.000474	0.002993	1
52.25	0.986843	0.000006	0.000929	0.005960	0
52.5	1.000000	0.000000	0.000000	0.000000	1
52.75	0.993160	0.000000	0.000453	0.003136	0
53	1.000000	0.000000	0.000000	0.000000	1
53.25	0.992974	0.000000	0.000438	0.003234	0
53.5	1.000000	0.000000	0.000000	0.000000	1
53.75	0.992780	0.000000	0.000422	0.003336	0
54	1.000000	0.000000	0.000000	0.000000	1
54.25	0.992580	0.000000	0.000405	0.003441	0
54.5	1.000000	0.000000	0.000000	0.000000	1
54.75	0.992373	0.000000	0.000388	0.003551	0
Screen annually thereafter, until this individual starts treatment at age 67					
55	1.000000	0.000000	0.000000	0.000000	1
55.25	0.992159	0.000000	0.000370	0.003663	0
55.5	1.000000	0.000000	0.000000	0.000000	1
55.75	0.991938	0.000000	0.000352	0.003780	0
56	1.000000	0.000000	0.000000	0.000000	1
56.25	0.991708	0.000000	0.000332	0.003901	0
56.5	1.000000	0.000000	0.000000	0.000000	1
56.75	0.991471	0.000000	0.000312	0.004026	0
57	1.000000	0.000000	0.000000	0.000000	1
57.25	0.991225	0.000000	0.000291	0.004156	0
57.5	1.000000	0.000000	0.000000	0.000000	1
57.75	0.990971	0.000000	0.000270	0.004289	0
58	1.000000	0.000000	0.000000	0.000000	1
58.25	0.990709	0.000000	0.000247	0.004428	0
58.5	1.000000	0.000000	0.000000	0.000000	1
58.75	0.990437	0.000000	0.000223	0.004571	0
59	1.000000	0.000000	0.000000	0.000000	1
59.25	0.990156	0.000000	0.000199	0.004719	0
59.5	1.000000	0.000000	0.000000	0.000000	1
59.75	0.989865	0.000000	0.000174	0.004872	0
60	1.000000	0.000000	0.000000	0.000000	1
60.25	0.989564	0.000000	0.000147	0.005031	0
60.5	1.000000	0.000000	0.000000	0.000000	1
60.75	0.989253	0.000000	0.000120	0.005195	0
61	1.000000	0.000000	0.000000	0.000000	1
61.25	0.988931	0.000000	0.000091	0.005364	0
61.5	1.000000	0.000000	0.000000	0.000000	1
61.75	0.988598	0.000000	0.000062	0.005539	0
62	1.000000	0.000000	0.000000	0.000000	1
Screen annually thereafter, until this individual starts treatment at age 67					
